# Functional Amyloid Signaling via the Inflammasome, Necrosome, and Signalosome: New Therapeutic Targets in Heart Failure

**DOI:** 10.3389/fcvm.2015.00025

**Published:** 2015-05-19

**Authors:** Traci L. Parry, Jason H. Melehani, Mark J. Ranek, Monte S. Willis

**Affiliations:** ^1^McAllister Heart Institute, University of North Carolina, Chapel Hill, NC, USA; ^2^Department of Pharmacology, University of North Carolina, Chapel Hill, NC, USA; ^3^Section of Cardiology, Department of Medicine, The Institute for CardioScience, Johns Hopkins Medical Institutes, Baltimore, MD, USA; ^4^Department of Pathology and Laboratory Medicine, University of North Carolina, Chapel Hill, NC, USA

**Keywords:** functional amyloid, inflammasome, necrosome, signalosome, pharmacological chaperones

## Abstract

As the most common cause of death and disability, globally, heart disease remains an incompletely understood enigma. A growing number of cardiac diseases are being characterized by the presence of misfolded proteins underlying their pathophysiology, including cardiac amyloidosis and dilated cardiomyopathy (DCM). At least nine precursor proteins have been implicated in the development of cardiac amyloidosis, most commonly caused by multiple myeloma light chain disease and disease-causing mutant or wildtype transthyretin (TTR). Similarly, aggregates with PSEN1 and COFILIN-2 have been identified in up to one-third of idiopathic DCM cases studied, indicating the potential predominance of misfolded proteins in heart failure. In this review, we present recent evidence linking misfolded proteins mechanistically with heart failure and present multiple lines of new therapeutic approaches that target the prevention of misfolded proteins in cardiac TTR amyloid disease. These include multiple small molecule pharmacological chaperones now in clinical trials designed specifically to support TTR folding by rational design, such as tafamidis, and chaperones previously developed for other purposes, such as doxycycline and tauroursodeoxycholic acid. Last, we present newly discovered non-pathological “functional” amyloid structures, such as the inflammasome and necrosome signaling complexes, which can be activated directly by amyloid. These may represent future targets to successfully attenuate amyloid-induced proteotoxicity in heart failure, as the inflammasome, for example, is being therapeutically inhibited experimentally in autoimmune disease. Together, these studies demonstrate multiple novel points in which new therapies may be used to primarily prevent misfolded proteins or to inhibit their downstream amyloid-mediated effectors, such as the inflammasome, to prevent proteotoxicity in heart failure.

## Introduction

As the most common cause of death and disability globally, heart disease remains an incompletely understood enigma. Despite vast improvements in our understanding of the neurohormonal basis of heart failure, current therapies targeting this system have reached a plateau for which new therapeutic paradigms might be considered. A great deal of insight has recently been achieved by our understanding of the role of protein homeostasis in the heart. The role of misfolded proteins, once reserved as the pivotal pathophysiological mechanism in neurodegenerative diseases, such as Alzheimer’s disease, Huntington’s disease, and Parkinson’s disease, has now been demonstrated to be present in many types of heart failure.

## Misfolded Proteins in Cardiac Disease

A growing number of cardiac diseases are characterized by misfolded proteins underlying their pathophysiology, including cardiac amyloidosis and dilated cardiomyopathy (DCM). Cardiac amyloidosis represents a broad disease entity characterized by misfolded proteins forming insoluble aggregates that are deposited in the heart. While the physical morphology is shared by all amyloid fibrils, the spectrum of affected organs and clinical presentation and prognosis varies greatly. The prognosis is often dependent upon the makeup of the amyloid precursor protein and its associated pathophysiology throughout the body. At least nine precursor proteins have been implicated in the development of cardiac amyloidosis (1). The most common cause of cardiac amyloidosis is multiple myeloma (MM), specifically MM-secreted monoclonal Ig light chain disease (1), followed by disease-causing mutant or wildtype transthyretin (TTR) protein (2). Functionally, infiltrative amyloid deposition causes a restrictive cardiomyopathy (diastolic dysfunction) and subsequent systolic heart failure.

The pathophysiology of amyloid light-chain amyloidosis (AL) (Ig light chain) cardiomyopathy was hypothesized to be due to the extracellular deposition of fibrils, resulting in increased passive stiffness and loss of cardiac parenchyma due to cell death (1). The physiological impairment in diastolic filling in amyloid cardiomyopathy patients supported this idea (3, 4). However, evidence for precursor toxicity has gained traction with recent correlations of AL cardiomyopathy with ATTR cardiomyopathy function with similar degrees of fibril deposition (5). Surprisingly, patients with equal fibril deposition due to AL cardiomyopathy are functionally worse than ATTR patients indicating that the infiltration itself is not completely responsible for the dysfunction (5). Soluble factors found circulating in AL patients may be an additional contributor to the cardiac dysfunction. This has been shown in studies where experimental infusion of amyloidogenic light chain proteins from patients with severe cardiac involvement into healthy hearts. The infusion of amyloidogenic light chain proteins induced diastolic dysfunction in healthy unaffected isolated mouse hearts, while infusion of light chains from patients with myeloma and no amyloidosis do not (6–9). The cardiac dysfunction in amyloid cardiomyopathy therefore appears to be related to mechanisms induced by both infiltrative insoluble factors in the heart itself and circulating soluble factors from the systemic MM.

Recognition of protein aggregation as a cause of heart failure is likely underestimated. Two recent studies illustrate the prevalence of protein aggregation as a cause of heart failure in patients that had heart failure clinically characterized by DCM. Two new sequence variants in the presenilin-1 (PSEN1) gene promoter were identified in nearly 1% (3/325) of patients of the idiopathic DCM patients investigated (10). Interestingly, PSEN1 mutant patients have cardiac protein aggregates present, along with reduced *psen1* gene and PSEN1 protein expression (10). Mechanistically, PSEN1 co-immunoprecipitates with SERCA2a illustrating one point in which PSEN1 may be affecting cardiac function (10). With the PSEN1 oligomer interacting directly with the Ca2+ channel, it is possible that changes in Ca2+ and heart failure seen in these patients may be mechanistically linked by this interaction (10). Similarly, tangles and plaque-like aggregates made of COFILIN-2 have been found in other DCM cases, estimated to involve nearly one-third of the cases (11). Initial studies investigated the aggregate composition of aggregates extracted from human idiopathic DCM with Congo red positivity has been found to include COFILIN-2 in a high percentage of patients, which was confirmed in a larger cohort of samples (11). Aggregates had COFILIN-2 present, an actin-depolymerizing protein known to participate in neurodegenerative diseases (12, 13). Understanding COFILIN-2’s role in chronic degenerative diseases such as DCM offers a novel therapeutic target (11).

Mutations in heat shock proteins (HSPs), a critical component of the cellular “anti-folding” apparatus, also underlie human cardiac disease. HSP proteins assist protein folding in routine maintenance of the cardiomyocyte. However, in the context of disease, their recruitment to protein misfolding is critical with acquired conditions such as ischemia/reperfusion injury, or because of mutations which can modify protein structures (14). In Long QT Syndrome 2, mutations in KCNH2 (aka human ether-a-go-go related gene/HERG) encoding the rapidly activating-delayed rectifier potassium channel Kv11.1 alpha-subunit alter cell repolarization of the ventricular action potential (15). Characterized by prolonged QT interval and ventricular tachycardia, syncope, and sudden death, the largest number of HERG mutations (28/34) affect protein folding and trafficking (16). Similarly, the desmin contractile apparatus linking nucleus, mitochondria, and sarcolemma is critical to cardiomyocyte function. Desmin deficiency or mutations in the chaperone proteins assisting desmin folding, e.g., HSPA/HSP70, HSPH (HSP110), DNAJ (HSP40), HSPB (small HSPs), SHPD, HSPE, CCT, result in proteotoxicity mediated via aggregate formation (14).

## Protein Folding, Preamyloid Oligomers, and Aggregation

**Figure 1:**
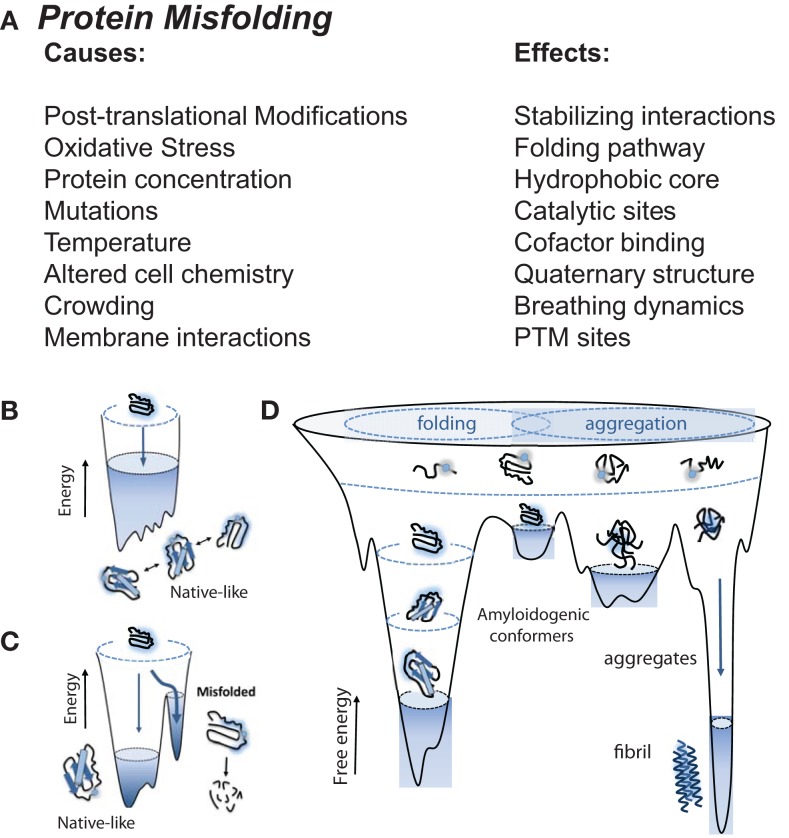
**Native, non-native, aggregates, and amyloid protein structures, and the stressors that drive them**. **(A)** Proteins are prone to misfolding by direct biological and indirect environmental stresses, including alterations in the protein sequence (mutations) and post-translational modifications (e.g., those induced by oxidative stress), respectively, creating protein aggregates and amyloid. These toxic structures are dangerous to biological systems, driving amyloidosis in neurodegenerative and cardiac pathologies. **(B)** Unfolded protein resides at a high entropy state in an unstable “non-native” structure. As they become folded, they move toward a lower entropy and move toward stable and favorable “native” structure. **(C)** Biological and environmental stressors initiate alterations in protein sequence (i.e., mutations), forcing intermediates into unstable conformations. **(D)** Destabilized proteins accumulate resulting into misfolded protein aggregates and amyloid with more stable conformations. **(B–D):** © Bentham Science. Used with permission from Gomes (20).

In biological systems, multiple physical factors influence protein folding (17), including mutations, molecular chaperones, and protein quality control systems (such as the ubiquitin proteasome system), which prevent the formation of misfit conformations resulting from destabilized protein folding and/or aggregate formation (18, 19). Protein misfolding is driven by alterations in the protein sequence (i.e., mutations), malignant post-translational modifications, and oxidative stress among other environmental cues (Figure 1A). These alterations initiate pathology through: (1) formation of a destabilized protein; (2) accumulation of intermediates with unstable folding, and (3) stabilization of misfolded protein conformations through the formation of aggregates (Figures 1B–D). While native conformation stability is characterized as having achieved the lowest free energy state, this feature may also explain the stability of aggregate/fibril formation in diseases, including heart failure in amyloidosis and non-amyloidosis-related states.

Protein folding is a process through which polypeptide chains wrap themselves to achieve optimal stability in their physiological environment (Figure 1B) (21). This process results from the lower energy “native-like” interactions that are more stable than “non-native-like” conformations (Figure 1C) (20). As favorable interactions are established, proteins transition from a high entropy state to one with a lower entropy structure. Proteins therefore have a range of free energies they can move through, inevitably proceeding through a set of conformations that narrow as the native state is achieved (22–24). Typically for small globular proteins, this transition has been likened to that of a funnel, where inter- and intra-domain interactions cooperatively interact to establish a stable native confirmation (25). Adverse conditions or mutations result in a degeneration of the energetic minimum, resulting in the lowering of barriers between native-like conformations (Figure 1D). The resulting larger distribution results in higher energy amyloidogenic conformers in destabilized folding patterns, susceptible to degradation or consequent local unfolding that triggers aggregation. These conformers can then contribute to the assembly into amorphous organized aggregates into fibrils results from interactions between stabilized aggregates (Figure 1D).

## Protein Aggregates, Proteotoxicity, and Heart Failure

**Figure 2:**
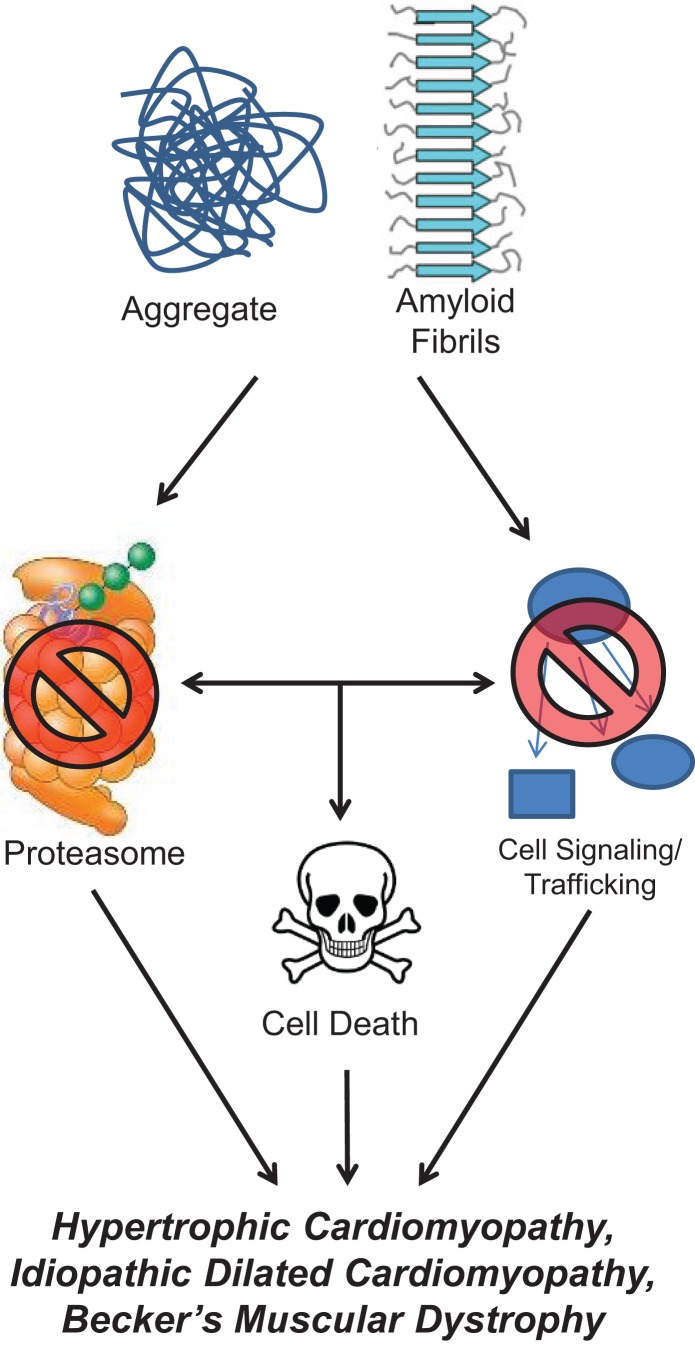
**Protein aggregate and amyloid stimulate cellular dysfunction**. Misfolded protein aggregates and amyloid inhibit normal proteasome function, interfere with cellular signaling and trafficking, and induce apoptotic cell signaling pathways. All of these perturbations have been shown to occur in cardiac pathologies, such as hypertrophic cardiomyopathy, idiopathic dilated cardiomyopathy, and Becker’s muscular dystrophy.

Protein aggregates and their preceding intermediates induced cell death is a process described as proteotoxicity (Figure 2). These designations refer to a protein’s state, not its origin as many different types of proteins can form these misfolded proteins. However, most familiar examples of aggregates tend to be recognized for their specific sources, such as β-amyloid (AB) and α-synuclein found in Alzheimer’s and Parkinson’s disease, respectively. Misfolded protein aggregation leading to heart failure has been found to be caused by misfolded TTR and excessive immunoglobulin resulting from aging or mutations and MM, respectively. Soluble oligomers and aggregated proteins disrupt cellular function by interfering with proteasome function, disrupting cell signaling, and protein trafficking, and directly support signals that induce cell death (26, 27). The accumulation of misfolded protein oligomers have been identified in patients with hypertrophic cardiomyopathy, idiopathic DCM, and Becker’s muscular dystrophy, but not in healthy controls (28). In DCM, cytosolic aggregates colocalize with ubiquitin and increased autophagy has been identified (29). Autophagy, a mechanism for removing aggregated proteins, may be one way in which the heart clears the pathological buildup of misfolded proteins.

The link between misfolded proteins, proteotoxicity, and heart failure has just recently been made. Early correlations between misfolded proteins and heart disease were made in mouse models of cardiac hypertrophy, where aggregation of misfolded proteins and aggresomes were identified (30). Further, inhibiting proteasome activity resulted in the accumulation of ubiquitinated protein aggregates and enhanced autophagy (30). However, it was not until the toxic effects of misfolded proteins in the heart were tested directly that the causal link of misfolded proteins to proteotoxicity and heart failure was made. In these studies, mouse hearts expressing small oligomers prone to misfolding (poly-Q83) were compared to hearts expressing a similar but non-oligomer forming peptide repeat (poly-Q19) (31). Mice with cardiac PQ83 developed heart failure by 5 months of age, whereas the non-aggregating control PQ19’s heart function was completely normal (31). These studies develop a new paradigm by which to consider how protein aggregation may contribute to heart failure and ischemic heart disease (32, 33), as well as genetic diseases associated with protein misfolding and cardiac dysfunction.

## How Endogenous Systems Prevent Protein Misfolding

Cells have adapted mechanisms to counteract the propensity of a protein to misfold, particularly in the face of common cellular challenges, such as oxidative stress, and the routine post-translational modifications that can accumulate on proteins. The ubiquitous HSPs act as molecular chaperones to counteract the stress-induced denaturation of other proteins. In the heart, the cardio-protective effect of HSP90, HSP70, TRiC, as well as a host of small HSPs [αBCrystallin (aka HSPB5), HSP20 (aka HSPB6), HSP22 (aka HSPB8, H11 kinase, and αCCrystallin), HSP27 (aka HSPB1 and HSPB2), and HSP60], has been described in the context of cardiac hypertrophy, heart failure, and ischemia reperfusion disease (34). Their ability to protect the misfolding of specific targets, such as actin, tubulin, desmin, lipid membranes, and mediators of cell death (e.g., Bax/Bak) have been implicated to date, though a thorough description of their role is just beginning to be elucidated (34).

Not only do chaperones play a role in maintaining proper protein folding, they have overlapping functions that might be useful when thinking about new therapeutic interventions in targeting protein misfolding. HSPs have overlapping functions – the integrity of a single protein can be maintained by >1 HSP in most cases. Recent studies have provided a proof of concept of how this knowledge may be applied therapeutically. Briefly, cultured cardiac cells with disease causing mutations in CryAB mediate cell death and dysfunction; as the complementary HSPB1 is introduced into the cells, both the aggregate formation and associated toxicity are relieved (35). As a consequence of this relationship, overexpressing HSPB1 facilitates the ubiquitination and proteasome degradation of protein aggregates induced by multiple CryAB misfolding mutations (35), illustrating that increasing HSP expression can help enhance both the solubility and degradation of misfolded proteins in cardiomyocytes. Similarly, treatment of the CryAB^R120G^ mutant mouse model of desmin-related cardiomyopathy with the drug geranylgeranyl-acetone (increasing HSPB8 and HSPB1) significantly reduces heart failure (36). CryAB^R120G^ transgenic mice treated with geranylgeranyl-acetone have reduced amyloid oligomer formation, decreased fibrosis, and recovery of heart function, and overall survival (36).

## Pharmacological Chaperone Therapies

Establishing the role of misfolded proteins in heart failure gives context to the emerging development of small molecule pharmacological chaperones (37–39). Pharmacological chaperones are defined as molecules which act specifically on misfolded or destabilized protein substrate(s) to specifically refold or stabilize these proteins, respectively (40). Pharmacological stabilization of the protein TTR and amyloid fibril disruptors are being developed for polyneuropathy. More recently, their applicability to amyloid disease in the heart has been demonstrated, and they are now undergoing clinical trials for cardiac amyloidosis due to misfolded TTR. Mutations and aging cause misfolding to result in TTR amyloidosis, and pharmacological chaperone therapy has successfully been used to treat both forms.

Initial studies investigating pharmacological chaperone therapy focused on the neurological manifestations of TTR amyloidosis. Extracellular deposition of TTR is a hallmark of the fatal autosomal dominant neurodegenerative familial amyloidotic polyneuropathy (FAP) (41), in which both mutant (familial forms) and wild type (senile/aging forms) aggregate. More than 80 different mutations destabilize TTR and prevent formation of the tetramer and promote aggregation of misfolded monomers (42). Misfolded TTR, also known as pre-albumin, is a protein synthesized by the liver that forms a soluble homotetramer, which acts to transport thyroid hormone (T4) and retinol. Extensive screening in aggregate-prone conditions led to the discovery of multiple aromatic small molecules that stabilize mutant TTR (43). These molecules, including specific NSAIDs and naturally derived flavonoid and xanthones, function by binding to thyroxine-binding sites in TTR to stabilize its structure (43). This led to the development of a wide range of compounds from different structural families, including enzoxazoles (44, 45). Tafamidis, a derivative enzoxazole, was selected for clinical development based on its ability to stabilize mutant TTR *in vitro*, and was later found to have a therapeutic effect in neurological disease models (46, 47). While occupying the thyroxine-binding sites, tafamidis’s negative cooperativity acts to stabilize the TTR tetramer.

The first human study of tafamidis for the treatment of polyneuropathy associated with TTR mutations was reported in 2012. Patients with the V30M TTR mutation and FAP were treated with 20 mg daily for 18 months (47). The primary objective was to monitor progression of neuropathy and to evaluate drug safety, while a secondary objective was to determine if tafamidis exhibits a stabilizing effect on human V30M TTR. A total of 128 patients were randomized to tafamidis or placebo. Significant reduction in neuropathy impairment scores were identified as early as 6 months and continued through 18 months (47). The changes in small nerve fiber function scores were attenuated by tafamidis, while allowing significant increases in BMI to occur (reflecting less muscle mass loss/atrophy resulting from the disease process) (47). Oral tafamidis is now approved in the EU for the treatment of TTR amyloidosis in adult patients with early stage symptomatic polyneuropathy to delay neurologic impairment. In Argentina, Japan, and Mexico, tafamidis is approved for use in delaying peripheral neurological impairment of TTR FAP (48). In the US, the FDA has requested a second efficacy study before it will grant approval, while noting that the data provided internal consistency and replication of effect.

In addition to neurodegeneration, patients with TTR-FAP develop cardiac disease. At least five clinical trials that investigate tafamidis in patients with TTR-related cardiomyopathy are active or have been completed (see Table 1). The first one recently published was a randomized crossover study in healthy volunteers that determined tafamidis did not have any effect on QTc intervals or serious adverse events in 42 subjects (49). Inhibition of TTR expression using siRNA therapy is yet another therapeutic approach being tested to treat TTR-related cardiomyopathy (see Table 1, rows 6–7).

**Table 1:** **Clinical studies investigating tafamidis and siRNA TTR in transthyretin-associated (amyloid) cardiomyopathy**.

1. TTR cardiomyopathy: clinicaltrials.gov NCT01775761 **Estimated enrollment: 42** **Estimated completion: 2013 (for primary outcome measure)**	A study to determine any effect of tafamidis on electrocardiographic intervals, specifically the rate corrected qt interval (qtc) *Endpoint: safety* **Phase 1:** ***randomized, placebo, and positive controlled cross-over study***	Completed/no study results posted **Primary outcome measures:** QTc interval using Fridericia’s correction method (QTcF) of tafamidis and placebo (baseline-adjusted) at each post-dose time **Secondary outcome measures:** QTcF of moxifloxacin and placebo at historical moxifloxacin median T_max_ of 3 h
2. TTR cardiomyopathy: clinicaltrials.gov NCT01655511 **Enrollment: 9** **Estimated completion: September 2012 (for primary outcome measure)**	Safety and pharmacokinetic assessment of orally administered tafamidis in healthy volunteers *Endpoint: pharmacokinetics of tafamidis >120 mg as an oral solution* **Phase 1:** ***randomized, double-blind, cross-over, ascending dose-tolerance***	Completed/no study results posted **Primary outcome measures:** safety and tolerability of orally administered tafamidis in healthy volunteers **Secondary outcome measures:** C_max_, T_max_, AUC 0-24, AUC last, AUC Inf, T_1/2_ plasma decay, TTR blood concentration, TTR stabilization
3. TTR-CM: clinicaltrials.gov NCT00925002 **Estimated enrollment: 110** **Estimated completion: 2021**	Safety and efficacy evaluation of fx-1006a in patients with v122i or wild-type transthyretin (ttr) amyloid cardiomyopathy *Endpoint: safety/efficacy* **Phase 3:** ***non-randomized, open-label evaluation of Tafamidis in patients with TTR amyloidosis***	Active, currently recruiting **Primary outcome measures:** long-term, open-label safety, and efficacy in ttr amyloidosis patients (10 years) **Secondary outcome measures:** provide investigation product until market availability to ATTR who have completed protocol
4. Cardiomyopathy: clinicaltrials.gov NCT00694161 **Estimated enrollment: 35** **Estimated completion: 2010 (final data collection for primary outcome measure)**	The effects of fx-1006a on transthyretin stabilization and clinical outcome measures in patients with v122i or wild-type ttr amyloid cardiomyopathy *Endpoint: safety/efficacy* **Phase 2:** ***interventional, open label, of ttr stabilization, and clinical outcomes in v122i or wildtype ttr amyloid cardiomyopathy***	**Completed (has results, below)** Primary outcomes: participants with TTR V122I and wildtype TTR achieved TTR stabilization at week 6 (97.1%, *N* = 35) Secondary outcomes: those achieving TTR stabilization continued through month 12 (87.5% with stabilized TTR, *N* = 34) Completed (has results, below)
5. Transthyretin (TTR) amyloid cardiomyopathy tafamidis: clinicaltrials.gov NCT01994889 **Estimated enrollment: 400** **Estimated completion: August 2018**	Tafamidis *Endpoint: safety/efficacy* **Phase 3:** ***multi-center, international, double-blind, placebo-controlled, randomized study to evaluate efficacy, safety, and tolerability of oral dosing of tafamidis meglumine in TTR-CM***	Recruiting, no study results posted **Primary outcome measures:** all-cause mortality and frequency of cv-related hospitalization **Secondary outcome measures:** 6 min walk test, Kansas City Cardiomyopathy Questionnaire, CV-related mortality, frequency of CV-related hospitalization, all-cause mortality, TTR stabilization at 1 month
6. TTR-mediated amyloidosis, ALN-TTRSC: clinicaltrials.gov NCT01814839 **Estimated enrollment: 76** **Estimated completion: 2015**	ALN-TTRSC (RNAi) *Endpoint: safety* **Phase 1:** ***randomized, double-blind, placebo-controlled, single and multi-dose, dose escalating study***	**Completed, no study results posted** **Primary outcome measures:** adverse events, serious adverse events, study drug discontinuation **Secondary outcome measures:** pharmacokinetics of ALN-TTRSC, effect of ALN-TTRSC on Vitamin A, effect of AL-TTRSC on retinol binding protein
7. TTR-mediated amyloidosis, ALN-TTRSC: clinicaltrials.gov NCT01981837 **Estimated enrollment: 25** **Estimated completion: January 2015**	ALN-TTRSC (RNAi) *Endpoint: safety/efficacy* **Phase 2:** ***open-label trial to evaluate safety, pharmacokinetics, pharmacodynamics, and Exploratory Clinical Trial of ALLN-TTRSC activity in TTR cardiac amyloidosis patients***	**Active, not recruiting, no study results posted** **Primary outcome measures:** AEs, SAEs, study drug discontinuation **Secondary outcome measures:** pharmacokinetics of ALN-TTRSC, effect of ALN-TTRSC on TTR

*TTR, transthyretin*.

*Created from data available at: clinicaltrials.gov (accessed 2 February, 2015)*.

Pfizer’s tafamidis and its potential to stabilize amyloid cardiomyopathy (TTR) are just the tip of the iceberg in the development of pharmacological chaperones for heart disease. Multiple other anti-amyloidogenic compounds have been discovered, including: (1) AG10, (2) doxycycline, (3) tauroursodeoxycholic acid (TUDCA), (4) (-)-epigallocatechin-3-gallate (EGCG), and (5) curcumin. High throughput screening for compounds that bind to the T4 pocket of TTR under physiological conditions identified AG10, which has higher affinity for TTR and greater pharmacokinetic stability while being more selective than tafamidis and diflunisal (a salicylic acid derivative) at physiological conditions (50). In *in vitro* studies, AG10 was found to effectively inhibit the proteotoxicity of V122I-TTR in human cardiomyocytes (50). AG10 inhibits aggregation of both wild-type TTR (associated with aging) and V122I-TTR significantly better than tafamidis, and almost fully stabilizes TTR at 10 μm plasma concentrations (42). AG10 is a bivalent molecule and thus able to bind 2 TTR proteins per molecule, allowing it to simultaneously occupy adjacent T4 sites in the TTR tetramer (42).

The discovery that tetracycline antibiotics have anti-amyloidogenic activity comes from the observation that iododoxorubicin (an anthracycline) is able to reduce amyloid and improve end organ damage in AL (51, 52). Doxycycline was then shown to act as a fibril disruptor *in vitro*, without toxicity (53). In mouse models of AL amyloidosis, doxycycline similarly disaggregated amyloid and improved tissue markers of TTR deposition, without effecting pre-fibril aggregates (54). In combination with the water-soluble bile acid TUDCA, synergistic clearance of TTR deposits was observed (55). Used in treating cholestasis in liver disease, TUDCA, like doxycycline, is an FDA approved drug for other indications. A phase 2, open-label study evaluating the efficacy, tolerability, safety, and pharmacokinetics of doxycycline and TUDCA in TTR-related cardiomyopathy is currently underway (Table 2).

**Table 2:** **Clinical studies investigating doxycycline in transthyretin-associated (amyloid) cardiomyopathy**.

8. Transthyretin amyloidosis, doxycycline + TUDCA: clinicaltrials.gov NCT01171859 **Estimated enrollment: 40** **Estimated completion: July 2015 (final data collection date for primary outcome measure)**	Doxycycline + TUDCA *Endpoint: safety/efficacy* **Phase 2:** ***single center, non-randomized, open-label, prospective study followed by 6 months withdrawal to evaluate efficacy, tolerability, safety, pharmacokinetics of TUDCA in TTR Amyloidosis***	Active, not recruiting **Primary outcome measures:** response rate to TUDCA (based in mBMI reduction <10%, change in neurologic impairment score-lower limbs (NIS-LL) <2, and NT-proBNP <30% (or <300 pg/ml) **Secondary outcome measures:** treatment-emergent adverse events, doxycycline PK, response in autonomic dysfunction, neuropathy and visceral organ involvement, incidence of patients discontinuing from study due to clinical or laboratory adverse events
9. Amyloidosis; heart (manifestation); senile cardiac amyloidosis; doxycycline + TUDCA: clinicaltrials.gov NCT01855360 **Estimated enrollment: 40** **Estimated completion: September 2015 (final data collection date for primary outcome measure)**	Doxycycline + TUDCA *Endpoint: safety/efficacy* **Phase 1/2:** ***open-label study of TUDCA in TTR-cardiomyopathy***	Recruiting **Primary outcome measures:** rate of progression of TTR cardiac amyloidosis (strain echocardiography) **Secondary outcome measures:** number of patients with adverse events to the medications over the period of therapy (18 months), evaluate general and health related QoL in senile and familial TTR amyloidosis subjects
10. Transthyretin amyloidosis; cardiomyopathy; doxycycline + UDCA: clinicaltrials.gov NCT02016365 **Estimated enrollment: 30** **Estimated completion: June 2015 (final data collection date for primary outcome measure)**	Doxycycline + UDCA *Endpoint: safety/efficacy* **Phase 2:** ***open-label study multi-center study of Safety and Efficacy of TUDCA in ATTR amyloidosis disease progression***	Active, not recruiting **Primary outcome measures:** efficacy on serum NT-proBNP (12 months) **Secondary outcome measures:** modified BMI, increase in septum thickness, neurologic Kumamoto Scale, number of patients with adverse events, blood work for potential drug-related adverse events

*TTR, Transthyretin; TUDCA,: tauroursodeoxycholic acid; UDCA, ursodeoxycholic acid*.

*Created from data available at: clinicaltrials.gov (accessed 2 February, 2015)*.

Additionally, there are a number of naturally occurring polyphenols that have anti-amyloidogenic properties, including the polyphenols EGCG and curcumin. EGCG, found in green tea, appears to bind amyloidogenic proteins and exert its protective effects by redirecting the aggregation process. Specifically, alternate non-pathogenic oligomeric species are instead formed which then deplete the formation of pathogenic types (56, 57). In a phase 2 clinical trial, EGCG was effective in preventing the progression of cardiomyopathy over 12 months. Of the 14 patients completing the study, no significant changes in cardiac mass or wall thickness were found when treated with ~500–700 mg EGCG daily (58). However, wide variation in bioavailability following oral EGCG intake is an issue complicating the clinical development of this compound (58, 59), including suspected hepatic toxicity (60). Still, at least three clinical trials evaluating EGCG for protein misfolding diseases are ongoing, two of which are in cardiac amyloidosis (Table 3). The other polyphenol, curcumin, competes for T4 on TTR to stabilize TTR’s native structure (61), inhibiting amyloid fibril formation *in vitro*, and disrupting pre-formed TTR fibrils from generating small oligomers (62). *In vivo* studies of transgenic TTR mice have demonstrated prevention of cytotoxicity and decreases in TTR deposition (63). However, there are no ongoing clinical trials evaluating curcumin for this therapeutic indication.

**Table 3:** **Clinical studies investigating ECGG in primary cardiac amyloidosis and Alzheimer’s disease**.

11. Light chain (AL) amyloidosis, cardiac involvement: clinicaltrials.gov NCT02015312**Estimated enrollment: 38Estimated completion: September 2017 (final data collection date for primary outcome measure)**	Green tea compound EGCG *Safety/efficacy* **Phase 2:** ***randomized, double-blind study of safety/efficacy***	Recruiting **Primary outcome measures:**12 months change in LV mass **Secondary outcome measures:**change in QoL, number of adverse events according to CTC criteria, change in cardiac biomarkers, hematological improvement, organ response (non-heart), overall survival
12. Alzheimer’s disease: clinicaltrials.gov NCT00951834 **Estimated enrollment: 50** **Estimated completion: June 2015 (final data collection date for primary outcome measure)**	Green tea compound epigallocatechin-3-gallete*Efficacy study***Phase 2/3:*randomized, double-blind study of efficacy***	Recruiting**Primary outcome measures:**ADAS-COG (score 0–70)**Secondary outcome measures:**safety and tolerability, MMSE score after 18 months vs. baseline, time to hospitalization and time to death related to AD, brain atrophy via MRI, baseline ADAS-COG and baseline-MMSE as covariates, CIBIC+ and WHO QoL Bref, trail making test, and MVGT
13. Primary (cardiac) amyloidosis of light chain type: clinicaltrials.gov NCT01511263 **Estimated enrollment: 86** **Estimated completion: December 2015 (final data collection date for primary outcome measure)**	Green tea compound epigallocatechin-3-gallete *Efficacy* **Phase 2:** ***open-label, randomized study of dietary EGCG***	Recruiting **Primary outcome measures:**cardiac response (rate of cardiac response following chemotherapy in patients with AL amyloidosis); endpoint at 6 months **Secondary outcome measures:**rate of adverse events, cardiac progression, time to cardiac progression, rate of cardiac events, time to cardiac events, survival at 6 months

*EGCG, epigallocatechin-3-gallete*.

*Created from data available at: clinicaltrials.gov (accessed 2 February, 2015)*.

Successful early studies of pharmacological chaperones in pre-clinical and now clinical trials of amyloid-based disease have supported the concept that reversing/stabilizing protein misfolding is possible and can attenuate and sometimes reverse the pathophysiology, even in the heart. The increasing number of clinical trials (both phase 1 and 2) with cardiac indication holds promise that we will better understand the efficacy and safety of these drugs in patients with heart failure soon. While specific pharmacological chaperones appear to be specific for their misfolded substrate (e.g., TTR), it will be interesting to see how applicable these chaperones are to other substrates, and whether pharmacological chaperones can be created for other substrate(s) found in differing causes of heart failure. With limited options for patients with heart failure, this class of drug offers a new way to treat an old but common problem.

Are pharmacological chaperones the only way to think about inhibiting proteotoxicity? It is possible that multiple points exist to prevent the proteotoxicity from contributing to heart failure, understanding that the formation of pre-amyloid oligomers, aggregates, and amyloids itself are a continuum. Like the concept of proteotoxicity itself, some of the answers may come from emerging discoveries in neurodegenerative diseases caused by endogenous misfolded proteins (e.g., Tau protein in Alzheimer’s disease) or exogenous proteins (e.g., caused by prions). Next, we introduce how the concept of altered proteostasis activates large signaling complexes, including the inflammasome and necrosome, as well as complexes to prevent misfolded protein-induced cell death (proteotoxicity), such as the signalosome.

## Amyloid Proteins, Heart Failure, and Activation of the Inflammasome

**Figure 3:**
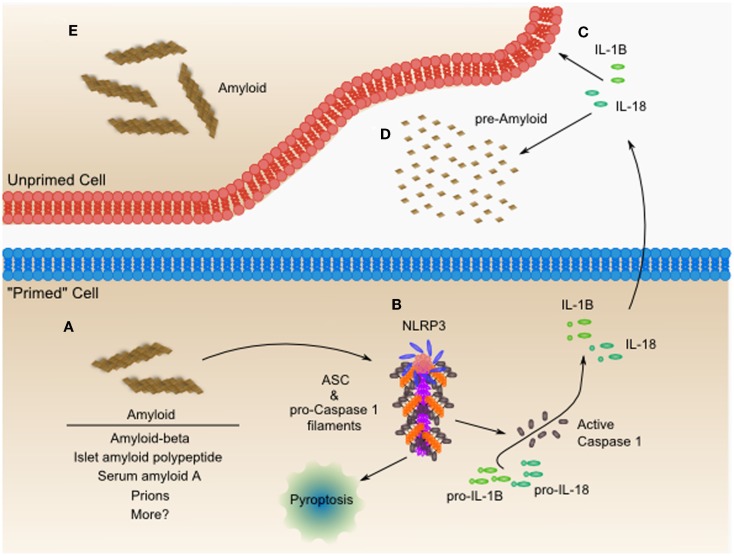
**The NLRP3 inflammasome is an amyloid-like fibrillar cytokine-processing platform, which senses amyloid and contributes to worsening heart failure**. **(A)** Amyloid fibrils from diverse sources including amyloid-beta, islet amyloid polypeptide, serum amyloid A, prions, and possibly others, accumulate within cells and tissues. **(B)** The NLRP3 inflammasome can activate in response to these amyloid fibrils, leading to formation of a functional amyloid nucleated by NLRP3 and containing long polymeric repeats of ASC, the adaptor protein, and pro-Caspase 1. The NLRP3 inflammasome is capable of inducing a pro-inflammatory necrotic cell death, termed pyroptosis. Additionally, the proximity of pro-Caspase 1 proteins to one another leads to their proteolytic activation and release of active Caspase 1. Active Caspase 1 then processes pro-IL-1β and pro-IL-18, among other proteins, leading to the secretion of the active form of these pro-inflammatory mediators. **(C)** Both IL-1β and IL-18 have been shown to directly cause cardiac dysfunction and may be targets for pharmacologic intervention as in the CANTOS trial. **(D)** IL-1β and IL-18 also can contribute to systemic inflammatory diseases characterized by massive production of acute phase reactants such as serum amyloid A. Serum amyloid A can then act as a seed for additional amyloid formation, which is sometimes seen in chronic inflammatory diseases such as Muckle-Wells syndrome (MWS) or rheumatoid arthritis (RA). In many resting cells, which do not express components of the NLRP3 inflammasome, these components can be upregulated through Toll-like receptor-NF-κB mediated signaling. Upon upregulation of inflammasome components, these cells are “primed” for inflammasome activation. It still remains to be seen whether inflammasome activation originating in the heart or from phagocytes drives the pathogenesis of heart failure. Additionally, studies have yet to tease out the role of NLRP3 inflammasome-mediated pyroptosis in cardiac dysfunction. **(E)** The inflammatory IL-1β and IL-18 may contribute to the formation of amyloid in neighboring cells, through unclear mechanisms. IL-18 co-localizes with Aβ-plaques and increases the hyperphosphorylation of tau-protein (72). IL-18 enhanced cleavage of serum amyloid-β precursor protein experimentally, and may be one mechanism of many remaining to be discovered.

The inflammasome is a large oligomeric signaling structure that generates a robust pro-inflammatory response through coordinated necrotic cell death and secretion of IL-1β and IL-18. While the composition varies based on the activating signal, the inflammasome generally is made up of three proteins, including a cytosolic sensory receptor, an adaptor protein ASC, and a Caspase 1 enzyme (64). The best characterized inflammasome, the NLRP3 inflammasome (Figure 3), is activated by a diverse array of infectious and sterile stimuli, which initiate signaling through perturbation of cellular homeostasis – either through mitochondrial disruption, ER stress, calcium influx, potassium efflux, or some combination of these mechanisms (65, 66). When activated, these proteins come together to form an enormous fibrillar structure that acts as a platform for cytokine processing (67–69). For example, NLRP3 mutations resulting in activation cause familial cold-induced inflammatory syndrome 1 (FCIS1), Muckle-Wells syndrome (MWS), and chronic neurologic cutaneous and articular syndrome (CINCA), all of which are characterized by hyperactive NLRP3 inflammasome signaling and chronic inflammation. Disruption of IL-1 signaling, with either the IL-1-receptor antagonist, Anakinra, or an anti-IL-1β monoclonal antibody, Canakinumab, provides rapid and dramatic resolution of symptoms (70, 71).

Some patients with activating mutations of NLRP3 develop multi-organ serum amyloid A protein (AA)-type amyloidosis – likely the result of persistently elevated levels of this acute phase protein in response to chronic inflammation (73). AA amyloidosis can involve deposits in the heart that lead to ventricular hypertrophy, atrial dilatation, and cardiac dysfunction (74–77). Although typically thought of as rare, a recent survey of re-examined autopsy tissues from 369 rheumatoid arthritis patients found that 30% had evidence of AA amyloid deposits with cardiac amyloid and renal amyloid appearing with equal frequency (78). In two recent case reports of AA amyloidosis, treatment with anti-cytokine therapies reduced chronic inflammation and serum amyloid A levels resulting in stabilized or improved cardiac manifestations of the disease (79, 80), providing further support for the hypothesis that limiting the availability of aggregation-prone proteins can limit amyloid formation.

While chronic inflammation can lead to amyloidosis, studies have also shown that the NLRP3 inflammasome can be activated in response to different types of amyloid, including amyloid-beta, islet amyloid polypeptide, serum amyloid A, prions, and curli fibers, a type of amyloid found in the biofilm of *Escherichia coli* and *Salmonella enterica* serovar Typhimurium (81–86). This diverse collection of amyloid that triggers the NLRP3 inflammasome raise the possibility that NLRP3 is central in generating an inflammatory response to *all* amyloids.

We are just beginning to appreciate the role of the NLRP3 inflammasome in cardiac disease. In mice, cardiac-specific overexpression of a constitutively active form of the calcineurin A catalytic subunit leads to impaired cardiac function (87) and myocardial inflammation characterized by activation of Caspase 1 (88). In this model, additional deletion of NLRP3 or pharmacologic inhibition of IL-1β signaling using the IL-1-receptor antagonist significantly reduced left ventricular dilatation and abrogated the progressive decrease in fractional shortening (FS) observed in wildtype and saline treated mice, thus implicating the NLRP3 inflammasome in this cardiac dysfunction (88).

In patients with idiopathic DCM, increased mRNA levels of NLRP3 inflammasome components were associated with worsening left ventricular ejection fraction, and NLRP3 and IL-1β mRNA at the time of admission were independent predictors of 6 months re-hospitalization (89). In support of a role for IL-1β signaling in cardiac dysfunction, human atrial trabecule tissue in an organ bath exhibited compromised systolic and diastolic function when exposed to TNF-α and IL-1β individually, and had additive effects when combined at low doses (90). Additionally, in patients with rheumatoid arthritis without concurrent cardiovascular disease, treatment with anakinra improved non-invasive measures of vascular and left ventricular function (91).

In 2011, a large multicenter trial called Canakinumab Anti-inflammatory Thrombosis Outcomes Study (CANTOS), which planned to enroll 17,200 post myocardial infarction patients, was initiated to determine whether IL-1β inhibition can prevent recurrent myocardial infarction, stroke, and cardiovascular death among stable patients with coronary artery disease who remain at high vascular risk (92). This trial will provide the most comprehensive clinical evaluation to date of the inflammatory hypothesis in cardiac disease.

These studies highlight the emerging and pivotal role that the NLRP3 inflammasome and IL-1β signaling play in the pathogenesis of heart failure. Continued interrogation of the NLRP3 inflammasome in heart failure is likely to aid in identifying new targets for therapeutic intervention, which may act synergistically with pharmacologic chaperones to break the cycle of amyloidosis and inflammation.

## Amyloid Proteins, Heart Failure, and Activation of “Functional Amyloid”: Necrosome

While amyloid has primarily been described as a key mediator of pathological processes, it has now been recognized that when certain proteins form amyloid, it is part of their physiological role. That is, they act as a molecular switch to promote environmental adaptation and act as a regulator of intracellular signaling (93). This includes the receptor-interacting protein kinases RIP1 and RIP3, which are found in the heart, and activate cell death by forming the necrosome (93). Activation and assembly resulted from their ability to form a fibrillar complex with amyloid properties, which was observed during *in vitro* and *in vivo* experimental conditions (94). The RIP homotypic interaction motifs (RHIMs) of RIP1 and RIP3 mediate the assembly of heterodimeric filamentous structures, which can trigger downstream signaling and cell death (94).

In the heart, myocardial ischemia induces RIP3 expression, which is sufficient to induce necroapoptosis (95). Conversely, RIP3^-/-^ mice challenged with MI had better function and less hypertrophy 30 days post- infarction, accompanied by decreased inflammatory response and decreased ROS production (95). Similarly, inhibiting RIP1 with the small molecule necrostatin-1 (Nec-1) prevents necrotic cell death in experimental models of cardiac ischemia (96). This model also displayed less adverse remodeling, evidenced by less dilation, preserved systolic function, and reduced inflammation (TNFα mRNA and ROS) (96). Taken together, these studies illustrate both the importance of the RIP1/RIP3-mediated necrosis in ischemia, offering a new direction for therapy to disrupt physiologic amyloid-like proteins that contribute to pathology (97).

## The COP9 Signalosome Enhances Protein Degradation to Reduce the Misfolded Protein Burden

**Figure 4:**
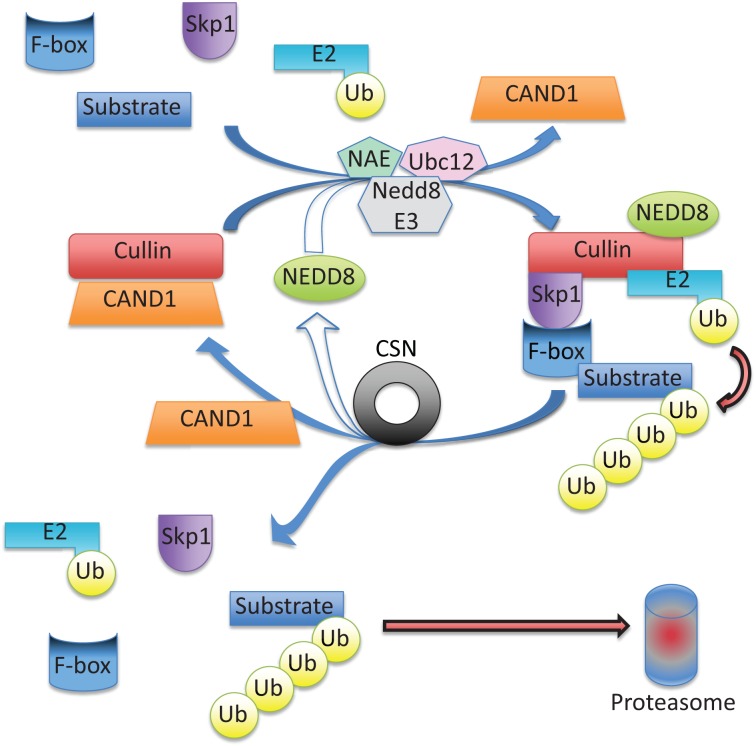
**Cullin-RING ubiquitin ligase (CRL) activity is regulated via neddylation and deneddylation**. Cullin serves as a scaffold protein when neddylated (conjugated with NEDD8), a reaction catalyzed by NEDD8 activating enzyme (NAE), ubiquitin conjugating enzyme (Ubc12), and a NEDD8-specific ligase (NEDD E3). Cullin neddylation triggers displacement of the inhibitory CAND1 (cullin-associated NEDD8-dissociated protein 1), allowing cullin to interact with the adapter protein, Skp1, and a F-box protein forming a functional CRL. Next, a ubiquitin charged E2 and a substrate are brought in close proximity to foster the transfer of ubiquitin to the substrate. After substrate ubiquitination, the COP9 Signalosome (CSN) will deneddylate (remove NEDD8) from cullin, which triggers the disassembly of the CRL, and frees the substrate to be degraded by the proteasome. The freed cullin can be neddylated again to initiate another cycle, or inhibited via CAND1 binding.

Enhanced protein degradation clinically can be attempted through blockade of the bad signaling complexes (e.g., the inflammasome and necrosome), or by activation of the beneficial constitutive photomorphogenesis mutant 9 (COP9) signalosome (CSN). Analogous to ubiquitination, proteins can be neddylated via a series of enzymatic reactions to conjugate NEDD8 to substrate proteins, such as a Cullin-RING-Ligase (CRL) (detailed in Figure 4), triggering the assembly of a functional CRL. CRLs are the largest family of ubiquitin ligases, including the ubiquitin ligases Parkin, Mdm2, Smurf1, and XIAP, in addition to transcription factors, such as E2F1, HIF1a, and p53 (98).

Only, recently has the role of deneddylation been explored. The CSN is a highly conserved multiprotein complex composed of eight subunits (CSN1–8) to form a ~350 kDa complex that deneddylates proteins (99) to regulate the protein degradation by ubiquitin proteasome system (100). CRL deneddylation is essential to disassemble the functional CRL and to release the ubiquitinated substrate protein for subsequent degradation by the proteasome. The CSN crystal structure has two organization centers: (1) a horseshoe-shaped ring created by its six PCI domain proteins and a large bundle formed by the carboxy-terminal α-helixes of each subunit (101). The CSN5-CSN6 dimer is found at the core of the helical bundle (101). Neddylated CRL binding to CSN is sensed by CSN4, communicated to CSN5 (with CSN6), resulting in activation of the deneddylase and regulation of downstream ubiquitin ligases (101).

Recent studies have demonstrated the COP9 signalosome regulates autophagosome maturation *in vivo*. Mice with a cardiomyocyte-specific deletion of *csn8* resulted in striking increases in autophagic flux (evidenced by increased LC3-II/LC3I, autophagosomes by TEM) and increased p62 protein (a functional amyloid forming protein) (102, 103). CSN appears to regulate Rab7, which plays a critical role in autophagosomal formation, and may explain its regulation of autophagy (102). Furthermore, the CSN appears to influence proteasomal activity by directly interacting with the 26S proteasome and potentially competing with the 19S proteasome lid (104, 105). Therefore, it is hypothesized that the overt loss of protein quality control due to compromised autophagosome formation and removal paired with reduced functionality of the UPS results in severe cardiomyocyte necrosis, leading to DCM, heart failure, and death.

Inducible cardiac CSN8^-/-^ mice demonstrated the role of CSN8 in regulating autophagic flux *in vivo*. The temporal cardiac Cre-LoxP ablation of CSN8 resulted in the accumulation of neddylated cullin (and non-cullin) proteins, increased ubiquitinated proteins, and a significant decrease in autophagic flux (103). Autophagosomes were markedly increased, as were oxidized proteins and necrotic cardiomyocytes, resulting in a dilated cardiomyopathic phenotype (103). How the COP9 signalosome is activated has not been clearly delineated. An intriguing possibility is that, similar to the proteasome, the COP9 signalosome can be regulated by post-translational modifications. Currently, there are no known proteins that post-translationally regulate the CSN; however, if found, this approach may have great therapeutic potential with small pharmacological modulators.

## Summary: The Structural Continuum between Functional and Pathological Amyloids and the Significance in Heart Failure

**Figure 5:**
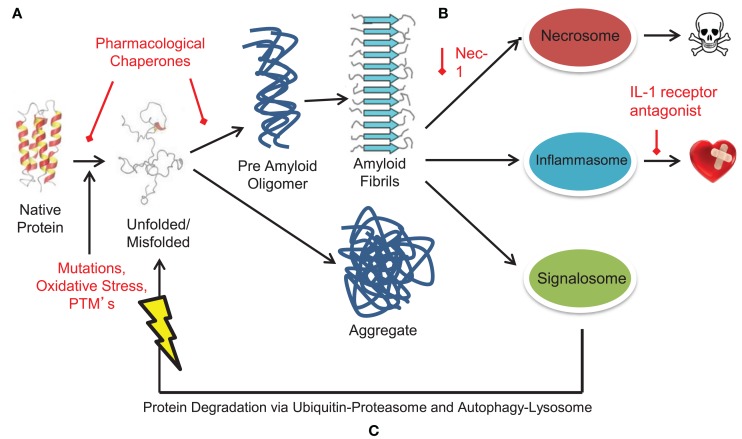
**Targeting pathological and functional aggregates in heart failure**. Alterations drive protein unfolding and misfolding, resulting in the formation of misfolded, toxic protein aggregates and amyloid. Such misfolded protein structures activate pathological pro-inflammatory and necrotic signaling complexes such as the inflammasome and necrosome. However, protein aggregates can also activate the signalosome to assist in clearing of mis/unfolded proteins. The continuum of protein misfolding to protein aggregation and amyloid formation to activation of large signaling complexes provides multiple levels for potential pharmacological therapeutic targeting. **(A)** Pharmacological chaperones target misfolded and unfolded proteins to stabilize protein conformation. Such drugs include tafamidis, doxycycline, and tauroursodeoxycholic acid. **(B)** Targeting and inhibiting large signaling structures like the inflammasome and necrosome offer new and possibly complementary methods of treating amyloid based diseases. To date, these targets have been inhibited through treatment with an IL-1 antagonist (inflammasome) and necrostatin-1 (necrosome). **(C)** Alternately, the COP9 signalosome may offer yet another therapeutic target to reduce the amyloidosis burden. Increasing activity of the signalosome could function alone or in tandem with other therapies to assist in clearance of misfolded protein aggregates and amyloid.

The formation of amyloids has been observed as a pathological change implicated in neurodegenerative disease, including Alzheimer’s disease, Parkinson’s disease, and polyglutamine proteins (Huntington’s disease). Paralleling these pathological findings and mechanisms, heart failure has similarly been found to involve these same biological processes (31). This has led to the multiple clinical studies underway testing pharmacological interventions to stabilize the common TTR misfolding in cardiac amyloidosis (Tables 1–3). However, the activation of amyloid formation is not only a pathological state; recent evidence suggests critical functional processes have similar aggregated states (Table 4) (93). Evidence that both the necrosome and inflammasome can be inhibited to demonstrate the utility of targeting their activation when aggregates have already formed, complementing the pharmacological chaperones that largely prevent misfolded proteins from forming and possibly reversing/removing protein aggregation. Complementary to these three pathways, activation of the COP9 signalosome by a yet to be determined pathway may prove to be another way in which misfolded cardiac proteins can be cleared to reduce future aggregate burdens. The multiple levels in which heart failure may be targeted illustrate many new opportunities for treating the heavy burden of heart failure where limited therapies exist, none of which currently target the underlying protein quality control issues outlined in this review (Figure 5).

**Table 4:** **Proteins forming amyloid as part of their functional role in cellular responses to the environment**.

Proteins	Species	Roles		Detection methods for aggregates^a^
		Soluble state	Aggregated state	
Sup35	Yeast	Translation terminator	Functional	ThT, CR, EM, X-ray, etc
Mod5	Yeast	tRNA isopentenyltransferase	Functional	ThT, EM
CPEB	Marine snail	Transcriptional activator/repressor	Functional	ThT, EM
	Fruit fly	
Pmel17	Human	Melanin synthesis	Functional	ThT, CR, EM, X-ray
RIP1, RIP3	Human	Kinases	Functional	ThT, CR, EM, X-ray
p62	Human	Sequestosome formation	Functional	ThT, EM
GW182	Human	P-body formation	Functional	EM
RCK/p54	Human	P-body formation	Functional	EM
TIA-1	Human	Stress granule formation	Functional	ThT, CR, EM
TDP-43	Human	DNA/RNA binding protein	Pathological/functional?	ThT, CR, EM
FUS/TLS	Human	DNA/RNA binding protein	Pathological/functional?	EM

*^a^ThT, thioflavin T; CR, congo red; EM, electron microscopy; X-ray, X-ray diffraction*.

*^©^2013 Elsevier Publishing. Used with permission from Furukawa and Nukina (93)*.

## Conflict of Interest Statement

The authors declare that the research was conducted in the absence of any commercial or financial relationships that could be construed as a potential conflict of interest.

## Abbreviations

AL, amyloid light-chain amyloidosis; CANTOS, Canakinumab Anti-inflammatory Thrombosis Outcomes Study; CINCA, chronic neurologic cutaneous and articular syndrome; COP9, constitutive photomorphogenesis mutant 9; CRL, cullin-RING-ligase; CSN, COP9 signalosome; EGCG, (-)-epigallocatechin-3-gallate; FAP, familial amyloidotic polyneuropathy; FCIS1, familial cold-induced inflammatory syndrome 1; HIF1α, hypoxia-inducible factor 1-alpha; HSP, heat shock protein; IL-1β, interleukin-1beta; Mdm2, mouse double minute 2 homolog; MWS, Muckle-Wells syndrome; Nec-1, necrostatin-1; NLRP3/NALP3, NACHT, LRR, and PYD domains-containing protein 3; Poly-Q19, poly-glutamine (19 repeats); Poly-Q83, poly-glutamine (83 repeats); p53, tumor protein 53; RA, rheumatoid arthritis; RHIMs, RIP homotypic interaction motifs; RIP1/RIP3, receptor-interacting protein 1/3; ROS, reactive oxygen species; TNFα, tumor necrosis factor alpha; TTR, transthyretin; TUDCA, tauroursodeoxycholic acid; XIAP, X-linked inhibitor of apoptosis protein.
